# Factors for Treatment Failure After Fecal Microbiota Transplantation in *Clostridioides difficile* Infection

**DOI:** 10.3390/microorganisms12122539

**Published:** 2024-12-09

**Authors:** Soo-Hyun Park, Jung-Hwan Lee, Suhjoon Lee, Jongbeom Shin, Boram Cha, Ji-Taek Hong, Kye Sook Kwon

**Affiliations:** 1 Department of Neurology, Soon Chun Hyang University Hospital Seoul, Seoul 05355, Republic of Korea; 2 Division of Gastroenterology, Department of Internal Medicine, Inha University Hospital, Inha University School of Medicine, Incheon 22332, Republic of Korea; suhjoon7@nate.com (S.L.); shinjongv@naver.com (J.S.); hahahjt2@naver.com (J.-T.H.);; 3 Department of Hospital Medicine, Inha University Hospital, Inha University School of Medicine, Incheon 22332, Republic of Korea

**Keywords:** fecal microbiota transplantation, microbiome, risk factor, outcome, antibiotics

## Abstract

Recently, fecal microbiota transplantation (FMT) has been introduced as an effective treatment option for *Clostridioides difficile* infection (CDI). However, the risk factors associated with FMT treatment failure have not been well demonstrated. Therefore, we aimed to investigate the risk factors of treatment failure or recurrence after FMT for CDI. This retrospective study included 124 patients with CDI who underwent FMT at Inha University Hospital between November 2017 and August 2021 and were followed up for 8 weeks after FMT for symptoms of CDI. FMT failure was defined as diarrhea recurrence or a positive stool test. We assessed the risk factors for treatment failure, including comorbidities, antibiotic use pre- and post-FMT, and the number of CDI episodes before FMT. Ninety-three patients (75%) experienced symptom improvement <7 days after FMT, while treatment failure occurred in 40 patients (32.3%). Multivariate analysis revealed that males had a lower symptom improvement rate <7 days after FMT (*p* = 0.049). Patients using antibiotics after FMT showed a higher rate of recurrence or treatment failure in <8 weeks (*p* = 0.032). Patients requiring antibiotics after FMT should be considered at higher risk of treatment failure. Careful antibiotic stewardship, particularly minimizing non-essential antibiotic use before and after FMT, may significantly enhance treatment outcomes. Further large-scale prospective studies are warranted to confirm these findings and develop targeted antibiotic management protocols for improving the efficacy of FMT in CDI treatment.

## 1. Introduction

Fecal microbiota transplantation (FMT) has emerged as a revolutionary treatment for recurrent and refractory *Clostridioides difficile* infections (CDI), providing a promising alternative for patients who do not respond to conventional antibiotic therapies [1]. FMT involves the infusion of processed stool from a healthy donor into the gastrointestinal tract of a patient to restore a balanced and healthy gut microbiota. This procedure has gained significant attention due to its high efficacy, with reported success rates in treating recurrent CDI ranging from 80% to 90% [1,2].

Despite the high overall success rate, a considerable proportion of patients experience FMT failure. FMT failure is typically defined as the recurrence of CDI symptoms or a positive stool test result for CDI within 8 weeks of the procedure [3]. Studies have indicated that the failure rates for FMT range from 20% to 30%, which differ according to the number of FMTs for multiple recurrent CDI [4,5]. Efficacy is influenced by several factors. Both advanced age and a higher Charlson Comorbidity Index (CCI) score have been linked to increased FMT failure rates, potentially due to weakened immune systems and impaired gut microbiota recovery in these patients [4,6]. The composition of the donor stool microbiome is crucial, with higher diversity leading to better engraftment and the restoration of healthy gut microbiota in the recipient, ultimately improving treatment success [7].

Although these factors have been identified, the existing literature on FMT failure is relatively sparse. This study aimed to address this by conducting a detailed analysis of the factors associated with FMT failure in patients with CDI. By examining a wide range of potential risk factors, including patient demographic and clinical characteristics, comorbidities, and patterns of antibiotic usage, this study seeks to provide a clearer and more comprehensive understanding of FMT efficacy prediction.

## 2. Materials and Methods

### 2.1. Patient Selection

This study employed a retrospective cohort design to investigate the risk factors associated with treatment failure after FMT for CDI. Data were collected from patients who underwent FMT at Inha University Hospital, Incheon, Korea, between March 2018 and July 2021. This study was reviewed and approved by the ethics committee of Inha University Hospital (Approval No. IUH 2024-10-040).

### 2.2. Data Collection

The inclusion criteria were adult patients diagnosed with CDI who underwent FMT, with the diagnosis confirmed by positive stool tests for *C. difficile* toxin B polymerase chain reaction (PCR) or glutamate dehydrogenase (GDH) with new-onset diarrhea [8]. Detailed patient information, including pre- and post-FMT data, was extracted from electronic medical records to comprehensively capture all relevant variables. Patients who underwent more than one FMT for CDI or those who could not be followed up for at least 8 weeks post-FMT were excluded from the study [8,9].

The variables collected encompassed several categories. Demographics included the median [interquartile range (IQR)] age of patients, as well as the distribution of male and female patients. The clinical characteristics recorded were diagnoses at admission, such as colitis or ileus, pneumonia, urinary tract infection, and other conditions. Comorbidities were also documented, including the presence of diabetes mellitus, liver disease, human immunodeficiency virus infection, malignancy (leukemia, lymphoma, localized solid tumor, and metastatic solid tumor), chronic kidney disease, congestive heart failure, acute myocardial infarction, peripheral artery occlusive disease, chronic obstructive pulmonary disease, cerebrovascular accident, hemiplegia, rheumatic disease, and dementia. The CCI was used to quantify patient comorbidities, adjusted for age [10].

### 2.3. Antibiotic Administration

Antibiotic usage was another critical variable, with data on the use of non-CDI antibiotics within one month prior to FMT and usage after FMT classified according to duration (≤7 and >7 days). The use of CDI-specific antibiotics, such as oral vancomycin and metronidazole, before and during FMT was also recorded. FMT outcomes were tracked, including the resolution of symptoms within seven days post-FMT and treatment failure, which was defined as diarrhea recurrence or a positive stool test for CDI within 8 weeks post-FMT [8,11]. This comprehensive data collection aimed to identify and analyze the risk factors associated with FMT failure in patients with CDI.

### 2.4. Statistical Analysis

Statistical analyses were conducted to evaluate risk factors for FMT treatment failure. Categorical variables are presented as frequencies and percentages, whereas continuous variables are summarized using medians and interquartile ranges (IQRs). Data analysis involved both univariate and multivariate approaches to identify potential risk factors for FMT failure. To explore potential risk factors for treatment failure, we conducted univariate analysis using Chi-squared or Fisher’s exact tests for categorical variables and Mann–Whitney U tests for continuous variables. Odds ratios (ORs) and 95% confidence intervals (CIs) were calculated to assess the strength of association between each variable and FMT treatment failure. Variables that were significant in the univariate analysis were subsequently included in the multivariate logistic regression model. This model was used to adjust for confounders and identify independent predictors of FMT failure, providing a more robust understanding of the factors contributing to treatment outcomes. All statistical tests were two-tailed, and a *p*-value < 0.05 was considered statistically significant. Data analyses were conducted using R software (version 4.2, R Foundation for Statistical Computing, Vienna, Austria).

## 3. Results

**Figure 1:**
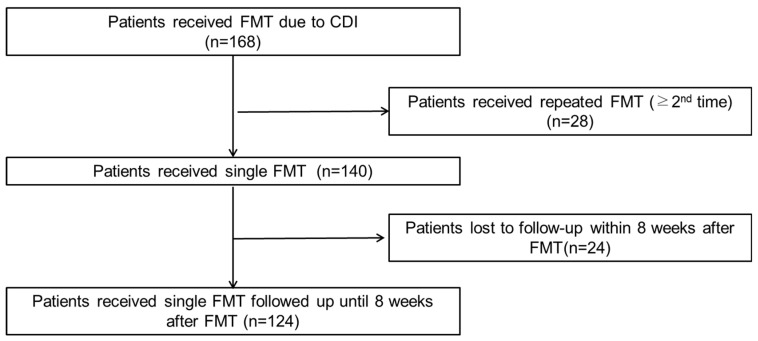
Flowchart of patient selection for the investigation of *Clostridium difficile* infection treatment failure in fecal microbiota transplantation.

Figure 1 presents the patient selection process for the study. It begins with the total number of patients who underwent FMT for CDI and outlines the criteria for inclusion and exclusion, with the final sample size for analysis highlighted. Patients excluded due to multiple FMTs or lack of follow-up are detailed. Initially, 168 patients received FMT, with 28 of these patients undergoing repeated FMT (≥2nd time). Of the remaining 140 patients who underwent a single FMT, 24 were lost to follow-up within 8 weeks post-FMT. Ultimately, 124 patients who underwent a single FMT were successfully followed-up for 8 weeks after the procedure.

As shown in Table 1, the study participants had a median (IQR) age of 75 (62–81) years and were fairly evenly split by sex, with 59 males (47.6%) and 65 females (52.4%). The most common diagnosis upon admission was colitis or ileus (92 patients, 74.2%), followed by pneumonia and urinary tract infections, each diagnosed in 11 patients (8.9% and 20.4%, respectively). Comorbidities were prevalent, with uncomplicated diabetes mellitus (25.0%), chronic kidney disease (21.8%), and dementia (18.5%) being the most frequent. Other notable conditions included cerebrovascular accidents (22.2%), hemiplegia (10.5%), and malignancies (8.1%). The median (IQR) age-adjusted CCI was 5 (3–6).

Table 2 summarizes the characteristics of antibiotic use and treatment outcomes in patients. Of the 124 patients included in this study, initial CDI diagnosis was made using GDH in 48 patients (38.7%), toxin B PCR in 16 patients (12.9%), and a combination of GDH and toxin B PCR in 60 patients (48.4%). Regarding antibiotic use, 79 patients (63.7%) received non-CDI antibiotics before FMT. After FMT, 43 patients (34.7%) received non-CDI antibiotics within seven days after FMT, whereas 53 patients (42.7%) received non-CDI antibiotics after seven days. Regarding CDI-specific treatments, 36 patients (29%) received CDI antibiotics for recurrent or refractory CDI before FMT, and 33 patients (26.6%) were treated with oral vancomycin during FMT, with a median duration of 13 days (IQR: 5–21). Colonoscopy was the most common method of FMT administration, used in 99 patients (79.8%), followed by duodenoscopy in 11 patients (8.9%), and a combination of both in 14 patients (11.3%). FMT outcomes were favorable, with 93 patients (75%) experiencing symptom resolution within seven days post-transplantation.

The analysis of antibiotic usage among patients revealed significant patterns in the types and frequencies of antibiotics administered before and after FMT (Table S1). Prior to FMT, the most frequently used non-CDI antibiotics were 2nd–3rd-generation cephalosporins (53.8% in patients with CDI recurrence vs. 38.8% in those without, *p* = 0.17), extended-spectrum penicillins (48.7% vs. 31.8%, *p* = 0.106), and quinolones (33.3% vs. 36.5%, *p* = 0.891). Post-FMT, aminoglycosides were significantly associated with CDI recurrence, being used in 25.6% of patients with recurrence compared to 7.1% in those without (*p* = 0.01). Similarly, aztreonam use post-FMT was notably higher in patients with recurrence (15.4% vs. 1.2%, *p* = 0.006).

The analysis of risk factors associated with FMT failure revealed several significant findings (Table 3). Prolonged use of non-CDI antibiotics (>7 days) after FMT was strongly associated with treatment failure, with 66.7% of patients experiencing failure compared to those without prolonged antibiotic use (OR: 4.30, 95% CI: 1.92–9.63, *p* < 0.01). This association remained significant in the multivariable analysis (OR: 2.62, 95% CI: 1.08–6.36, *p* = 0.03). Additionally, the use of non-CDI antibiotics before FMT was observed in 84.6% of patients with treatment failure, showing a significant association in both univariate (OR: 4.66, 95% CI: 1.77–12.29, *p* = 0.001) and multivariable analyses (OR: 3.03, 95% CI: 1.12–8.19, *p* = 0.03). Moreover, the administration of specific antibiotics such as aminoglycosides after FMT increased the likelihood of failure (OR: 4.54, 95% CI: 1.51–13.61, *p* = 0.007; multivariable OR: 3.69, 95% CI: 1.14–12.00, *p* = 0.03). Aztreonam showed a notably high OR in the univariate analysis (OR: 15.27, 95% CI: 1.77–131.77, *p* = 0.013); however, this was not statistically significant in the multivariable analysis (*p* = 0.06). Other variables, such as the use of non-CDI antibiotics within seven days after FMT, CDI antibiotics (oral vancomycin) during FMT, male sex, and age-related CCI, were not significantly associated with treatment failure in either the univariate or multivariate analyses.

## 4. Discussion

The findings of this study provide insight into the factors that influence FMT treatment success for CDI. Although a significant number of CDI diagnoses in this study were based solely on nucleic acid amplification tests (NAATs), most patients presented with symptoms of diarrhea sufficiently severe to warrant FMT [12,13]. In such cases, the clinical presentation, including persistent and debilitating diarrhea, was corroborated by colonoscopic findings, supporting the decision to proceed with FMT despite the limitations of NAATs in distinguishing colonization from active infection. In cases where oral vancomycin was used prior to FMT, the duration often exceeded 14 days, owing to the implementation of a tapering regimen [14]. The analysis revealed that the use of non-CDI antibiotics before and after FMT significantly increased the risk of treatment failure. This association underscores the importance of minimizing unnecessary antibiotic use to preserve FMT efficacy. The prolonged use of non-CDI antibiotics after FMT was found to be a strong predictor of treatment failure. This finding suggests that antibiotics, which can disrupt the gut microbiota, hinder the successful re-establishment of a healthy microbial community post-FMT [15]. Therefore, antibiotic therapy following FMT should be carefully considered, and non-essential antibiotics should be discontinued as soon as possible. Additionally, this study highlighted that the use of non-CDI antibiotics before FMT poses a significant risk. Antibiotic exposure before FMT might impair the initial establishment of donor microbiota, leading to higher rates of treatment failure [16]. These results align with previous research that identified antibiotic use as a critical factor affecting FMT outcomes [15,16,17]. These findings emphasize the need for antibiotic stewardship before and after FMT to enhance treatment efficacy.

Previous studies have highlighted various risk factors for FMT failure, including CDI severity, inpatient status at the time of FMT, and increased frequency of CDI-related hospitalizations [18]. In addition, factors such as advanced age, inflammatory bowel disease, and peri-FMT use of non-CDI antibiotics have been implicated in FMT failure [19,20]. Our study also demonstrated that antibiotic use before FMT plays a critical role in FMT efficacy. Previous studies have demonstrated caution in using antibiotics before and after FMT, coinciding with our current study [15,17,18]. However, few studies have investigated FMT failure based on prior antibiotic use. This explains why prior antibiotic therapy disrupts the recipient’s gut microbiota, making it harder for the transplanted microbes to eliminate themselves [21,22]. Our previous studies revealed that the degree of dysbiosis impacts the effect of FMT or probiotics on the clearance of multidrug-resistant organisms in feces [23,24]. The confirmation of non-CDI antibiotic use before FMT as an independent risk factor emphasizes the need for judicious antibiotic stewardship before and after FMT [25].

Furthermore, the use of antibiotics other than those targeting CDI after FMT can disrupt the newly introduced gut community and increase the risk of CDI recurrence [4,22]. The identification of prolonged non-CDI antibiotics post-FMT as a significant risk factor aligns with prior research but extends our understanding of the duration of antibiotic impact. In our study, non-CDI antibiotics administered >7 days after FMT were associated with a higher risk of CDI recurrence. It is possible that non-CDI antibiotics administered >7 days after FMT tended to be more prolonged than those administered <7 days after FMT. In addition, refractory and recurrent CDI after antibiotic treatment is a strong independent factor for FMT failure. Although there are few studies on the duration between CDI recurrence, severe CDI, prior CDI-related hospitalization, and inpatient status, these have been demonstrated to be significant predictors of FMT failure [19]. Recurrent CDI within one month might have met these conditions; therefore, it affected failure as a strong factor.

Aminoglycosides and aztreonam may have worse effects on FMT for CDI treatment owing to their broad-spectrum activity and potential to disrupt the newly transplanted microbiota. CDI is frequently associated with the use of aminoglycosides in combination with other antibiotics, which may exacerbate their detrimental effects on FMT efficacy [26]. Consequently, antibiotic regimens that include aminoglycosides may pose an increased risk to successful FMT outcomes for CDI. Similarly, aztreonam has been demonstrated to carry one of the highest risks for CDI among antibiotics [27]. Notably, clindamycin, which is widely recognized as having a high association with CDI development, was excluded from the current study because of its limited use under our hospital’s antibiotic stewardship policy during the study period. This underscores the critical importance of implementing robust antibiotic stewardship practices during the peri-FMT period to optimize outcomes. However, our study was limited in its ability to fully evaluate the effects of antibiotics on FMT due to an insufficient sample size. Further research with larger cohorts is needed to clarify these associations and inform clinical guidelines.

These findings have profound implications in clinical practice. First, the strong association between prolonged non-CDI antibiotic use after FMT and treatment failure suggests that clinicians should carefully evaluate the necessity of antibiotics after FMT and minimize their use whenever possible. This could involve the close monitoring of patients and implementation of antibiotic stewardship programs specifically tailored to patients receiving FMT [25,28]. Second, we found that non-CDI antibiotic use, especially before FMT, increased the risk of treatment failure, underscoring the importance of minimizing antibiotic exposure in the period leading up to FMT. Clinicians should consider strategies to reduce antibiotic use in patients scheduled for FMT, potentially by managing CDI with alternative treatments or using targeted narrow-spectrum antibiotics that are less likely to disrupt the gut microbiota before FMT. Third, this study was conducted in both inpatient and outpatient settings [29]. We investigated the negative effects of early antibiotic administration before and after FMT and demonstrated that prior antibiotics and post-antibiotics one week after FMT could contribute to FMT failure. Therefore, this study provides the appropriate timing for antibiotic use.

The future implications of this study center on improving CDI treatment outcomes through tailored antibiotic stewardship protocols and optimized FMT procedures [9,30]. By identifying non-essential antibiotic use, particularly before and after FMT, as a significant factor in treatment failure, this study suggests the need for targeted antibiotic stewardship protocols that carefully assess antibiotic necessity around the FMT period to preserve microbiota integrity. Additionally, the association between pre-FMT antibiotic exposure and FMT failure highlights the importance of developing clinical guidelines to manage patients with CDI who are candidates for FMT, offering a structured approach to optimize patient preparation and improve FMT success rates. Furthermore, considering patient-specific risk factors, such as comorbidities and extended post-FMT antibiotic use, indicates a future direction toward personalized FMT protocols, where individual risk profiles guide tailored pre- and post-FMT care. High-risk patients may benefit from enhanced microbiota monitoring and alternative therapies to support gut health, potentially improving outcomes for those with recurrent or refractory CDI. Future prospective, multi-center trials are necessary to generalize these findings and validate the conclusions across diverse patient populations and healthcare settings, thereby strengthening the foundation for widely applicable FMT guidelines and advancing CDI management.

### 4.1. Limitations

#### 4.1.1. Study Design Limitations

This study’s retrospective design introduces certain limitations, including potential selection bias. Retrospective studies inherently rely on previously recorded data, limiting control over variables and potentially impacting the reliability of causal inferences. Although we attempted to adjust for confounders, prospective studies are necessary to confirm these findings with greater control over variables.

#### 4.1.2. Single-Center Bias

As this study was conducted at a single institution, Inha University Hospital, the generalizability of our findings may be limited. Patient characteristics, treatment protocols, and outcomes may vary across different centers. Multi-center studies are necessary to validate our results across diverse populations and clinical settings.

#### 4.1.3. Data Collection Constraints

Data collection relied on electronic medical records, which may lack completeness and accuracy, potentially affecting the robustness of our analysis. Some relevant factors, such as dietary habits, probiotic use, or detailed donor microbiome characteristics, were not documented in the records and thus could not be analyzed. These unmeasured variables might have influenced FMT outcomes.

#### 4.1.4. Sample Size Considerations

The relatively small sample size (124 patients) may limit the statistical power of the study, particularly for variables with less frequent occurrence. A larger sample size would allow for more robust multivariable analyses and improve the generalizability of our findings. Future studies with a larger cohort are necessary to confirm our observations and better understand the factors influencing FMT success.

## 5. Conclusions

This study significantly advances the understanding of FMT failure in patients with CDI by identifying key risk factors and providing actionable insights for clinical practice. Minimizing antibiotic use before and after FMT represents a critical step toward improving treatment outcomes. By building on the existing body of research and highlighting areas for further investigation, this study lays the groundwork for more effective and targeted approaches to managing CDI with FMT.

## Figures and Tables

**Table 1:** Baseline clinical characteristics of patients who received fecal microbiota transplantation.

Characteristics	*n* (%)
Age, years [median (IQR)]	75 (62–81)
Sex	
Male	59 (47.6)
Female	65 (52.4)
Diagnosis at admission	
Colitis or ileus	92 (74.2)
Pneumonia	11 (8.9)
Urinary tract infection	11 (20.4)
Others	26 (21.0)
DM	
Uncomplicated DM	31 (25.0)
Complicated DM	3 (2.40)
Liver disease, mild	2 (1.60)
HIV	1 (0.8)
Malignancy	
Any leukemia, lymphoma, or localized solid tumor	8 (6.5)
Metastatic solid tumor	2 (1.6)
Chronic kidney disease	27 (21.8)
Congestive heart failure	11 (8.9)
AMI	3 (2.4)
PAOD	4 (3.2)
COPD	7 (5.6)
Cerebral vascular accident	12 (22.2)
Hemiplegia	13 (10.5)
Rheumatic disease	2 (1.6)
Dementia	23 (18.5)
Peptic ulcer disease	12 (9.7)
Age-adjusted Charlson Comorbidity Index	5 (3–6)

AMI: acute myocardial infarction; COPD: chronic obstructive pulmonary disease; DM: diabetes mellitus; HIV: human immunodeficiency virus; IQR: interquartile range; PAOD: peripheral occlusive arterial disease.

**Table 2:** *Clostridium difficile* infection and non-*Clostridium difficile* infection antibiotics characteristics and treatment outcomes.

Characteristics	*n* (%)
Initial CDI diagnosis	
GDH	48 (38.7)
Toxin B PCR	16 (12.9)
GDH + Toxin B PCR	60 (48.4)
FMT method	
Colonoscopy	99 (79.8)
Duodenoscopy	11 (8.9)
Colonoscopy + Duodenoscopy	14 (11.3)
Antibiotics	
Non-CDI antibiotics before FMT	79 (63.7)
Non-CDI antibiotics after FMT (≤7 days)	43 (34.7)
Non-CDI antibiotics after FMT (>7 days)	53 (42.7)
CDI antibiotics before FMT (recurrent or refractory CDI)	36 (29)
Oral vancomycin duration, median (interquartile range), days	13 (5–21)
Oral metronidazole duration, median (interquartile range), days	5 (3–9)
CDI antibiotics (oral vancomycin) during ongoing FMT	33 (26.6)
FMT Outcome	
Symptom resolution within 7 days after FMT	93 (75)

CDI: *Clostridium difficile* infection; FMT: fecal microbiota transplantation; PCR: polymerase chain reaction; GDH: glutamate dehydrogenase.

**Table 3:** Risk factors for fecal microbiota transplantation failure.

Variable	Treatment Failure	Univariate Analysis		Multivariable Analysis	
	n/total (%)	Odds ratio (95% CI)	*p*-value	Odds ratio (95% CI)	*p*-value
Non-CDI antibiotics after FMT (>7 days)	26/39 (66.7)	4.30 (1.92–9.63)	<0.01	2.62 (1.08–6.36)	0.03
Non-CDI antibiotics after FMT (≤7 days)	17/39 (43.6)	1.76 (0.80–3.84)	0.16		
Non-CDI antibiotics before FMT	33/39 (84.6)	4.66 (1.77–12.29)	0.001	3.03 (1.12–8.19)	0.03
Aminoglycoside after FMT	10/39 (25.6)	4.54 (1.51–13.61)	0.007	3.69 (1.14–12.00)	0.03
Aztreonam after FMT	6/39 (15.4)	15.27 (1.77–131.77)	0.013	8.28 (0.9–82.70)	0.06
CDI antibiotics (oral vancomycin) during FMT	13/39 (33.3)	1.63 (0.71–3.74)	0.253		
Male	18/39 (46.2)	0.92 (0.43–1.97)	0.829		
Age-related CCI		1.05 (0.91–1.21)	0.549		

CCI: Charlson Comorbidity Index; CDI: *Clostridium difficile* infection; CI: confidence interval; FMT: fecal microbiota transplantation.

## Data Availability

The dataset presented in this study is available from the corresponding author upon request.

## Supplementary Materials

The following supporting information can be downloaded at: https://www.mdpi.com/article/10.3390/microorganisms12122539/s1, Table S1: Non-CDI (*Clostridium difficile* infection) antibiotics before and after fecal microbiota transplantation.
