# Supplementary material for: Factors for Treatment Failure After Fecal Microbiota Transplantation in Clostridioides difficile Infection
Source: Microorganisms. 2024 Dec 9;12(12):2539. doi: 10.3390/microorganisms12122539 (PMC11677034; doi:10.3390/microorganisms12122539)
Supplement: Supplementary file 1 [file microorganisms-12-02539-s001.zip › microorganisms-3299403-supplementary.pdf]

Supplementary Table. Non-*Clostridium difficile* infection antibiotics before and after fecal microbiota transplantation

Variable n(%)	CDI recurrence (n=39)	No CDI recurrence (n=85)	*P*-value
Pre non-CDI antibiotics before FMT			
Aminoglycoside	7 (17.9)	8 (9.4)	0.29
Penicillin	1 (2.6)	4 (4.7)	0.943
Extend Spectrum Penicillin	19 (48.7)	27 (31.8)	0.106
Macrolide	1 (2.6)	1 (1.2)	>0.999
Carbapenem	3 (7.7)	6 (7.1)	>0.999
Glycopeptide	3 (7.7)	11 (12.9)	0.581
Cephalosphorin PO	1 (2.6)	2 (2.4)	>0.999
1th Cephalosphorin IV	3 (7.7)	8 (9.4)	>0.999
2-3th Cephalosphorin IV	21 (53.8)	33 (38.8)	0.17
Quinolone	13 (33.3)	31 (36.5)	0.891
Metronidazole	15 (38.5)	21 (24.7)	0.176
Azotreonam	2 (5.1)	3 (3.5)	>0.999

Colistimate	3 (7.7)	3 (3.5)	0.581
Tygecylcline	0 (0.0)	3 (3.5)	0.577
Trimethoprim-sulfamethoxazole	3 (7.7)	2 (2.4)	0.362
Rifamycin	3 (7.7)	3 (3.5)	0.581
Antifungal agent	0 (0.0)	4 (4.7)	0.407
Post non-CDI antibiotics after FMT			
Aminoglycoside	10 (25.6)	6 (7.1)	0.01
Penicillin	0 (0.0)	2 (2.4)	0.843
Extend Spectrum Penicillin	9 (23.1)	18 (21.2)	0.997
Macrolide	1 (2.6)	2 (2.4)	>0.999
Carbapenem	3 (7.7)	9 (10.6)	0.858
Glycopeptide	5 (12.8)	6 (7.1)	0.479
Cephalosphorin PO	0 (0.0)	1 (1.2)	>0.999
1th Cephalosphorin IV	2 (5.1)	3 (3.5)	>0.999
2-3th Cephalosphorin IV	10 (25.6)	18 (21.2)	0.748
Quinolone	9 (23.1)	12 (14.1)	0.328

Metronidazole	7 (17.9)	10 (11.8)	0.517
Azotreonam	6 (15.4)	1 (1.2)	0.006
Colistimate	2 (5.1)	0 (0.0)	0.181
Tygecylcline	4 (10.3)	2 (2.4)	0.146
Trimethoprim-sulfamethoxazole	4 (10.3)	4 (4.7)	0.439
Rifamycin	1 (2.6)	3 (3.5)	>0.999
Antifungal agent	2 (5.1)	5 (5.9)	>0.999

CDI: *Clostridium difficile* infection; FMT: fecal microbiota transplantation; PO: per os, which is Latin for “by mouth.”;IV: intravenous
